# Efficiency and obturation quality of Kedo-S rotary files versus hand files in pediatric endodontics

**DOI:** 10.6026/973206300223268

**Published:** 2026-06-30

**Authors:** Dinesh Solanki, Kundan Kumar Singh, Devendra Patil, Barkha Idnani, Rajendran Ganesh, Navsangeet Kaur Hans

**Affiliations:** 1 Department of Dentistry, JIET Medical College and Hospital, Jodhpur, Rajasthan, India; 2 Department of Conservative Dentistry & Endodontics, Rajendra institute of Medical Sciences, Ranchi, Jharkhand, India; 3 Department of Pediatric & Preventive Dentistry, TPCT's Terna Dental College, Navi Mumbai, Maharashtra, India; 4 Department of Conservative Dentistry & Endodontics, Faculty of Dental Science, Dharmsinh Desai University, Nadiad, Gujarat, India; 5 Department of Preventive Dental Sciences, Faculty of Dentistry, Al-Baha University, Al-Aqiq Campus, Kingdom of Saudi Arabia; 6 Department of Conservative Dentistry & Endodontics, Mangat Hospital, Ludhiana Chandigarh Highway, Katani Kalan, Ludhiana, Punjab, India

**Keywords:** Hand files, kedo-S rotary files, obturation quality, primary teeth, pulpectomy

## Abstract

Pulpectomy in primary teeth remains challenging due to time-consuming instrumentation and inconsistent obturation quality with conventional hand files. Therefore, it is of interest to compare the efficiency and obturation quality of Kedo-S rotary files with hand K-files in 100 primary molars of children aged 4-9 years. Instrumentation time and radiographic obturation quality (underfilled, optimally filled, overfilled and voids) were evaluated using appropriate statistical tests. Kedo-S rotary files showed significantly reduced instrumentation time, higher optimal fills (72% versus 52%) and fewer voids (18% versus 40%) compared to hand files (p <0.05). Thus, we show the clinical superiority of pediatric rotary systems in improving efficiency and obturation outcomes in pediatric endodontics.

## Background:

Pediatric endodontics plays a critical role in preserving the integrity and function of primary teeth until their natural exfoliation. Early childhood caries and traumatic dental injuries are among the most common causes of pulpal involvement in primary teeth, often necessitating pulpectomy procedures [1]. The primary goal of pulpectomy is the complete removal of necrotic or irreversibly inflamed pulp tissue, followed by through cleaning, shaping and obturation of the root canal system. Achieving optimal obturation is essential, as it directly influences the long-term success of the treatment by preventing reinfection and maintaining the tooth in a symptom-free state [2]. Primary teeth present unique anatomical challenges compared to permanent teeth. Their root canals are often tortuous, ribbon-shaped and exhibit numerous accessory canals [3]. Additionally, physiological root resorption and proximity to developing permanent tooth buds demand a high degree of precision during instrumentation and obturation. Traditional hand instrumentation techniques using stainless steel files have long been the standard approach in pediatric endodontics. While effective, these techniques are time-consuming and technique-sensitive and they may lead to procedural errors such as canal transportation, ledge formation and inadequate cleaning, especially in uncooperative pediatric patients [4]. In recent years, advancements in endodontic technology have led to the introduction of rotary instrumentation systems, which have revolutionized root canal preparation. Rotary files made from nickel-titanium (NiTi) alloys offer superior flexibility, shape memory and cutting efficiency compared to conventional stainless steel hand files [5]. These properties allow for more centered canal preparation, reduced procedural errors and improved shaping ability. In pediatric dentistry, where patient cooperation and chairside time are critical factors, rotary instrumentation offers the added advantage of significantly reducing treatment duration [6]. Among the various rotary systems developed specifically for primary teeth, Kedo-S rotary files have gained considerable attention. These files are uniquely designed with variable taper and length to suit the morphology of primary root canals [7]. Their shorter working length and pediatric-specific design enhance accessibility and ease of use in smaller oral cavities. Kedo-S files are also engineered to provide efficient debris removal while maintaining the original canal anatomy, thereby facilitating better obturation outcomes. The use of such specialized rotary systems has the potential to improve both the quality and consistency of endodontic treatment in children [8].

The comparison between Kedo-S rotary files and conventional hand files is particularly relevant in pediatric endodontics. While hand files are widely available and cost-effective, they may not always provide the same level of efficiency and consistency as rotary systems [9]. On the other hand, Kedo-S rotary files promise enhanced performance tailored to the unique anatomy of primary teeth. Evaluating these two approaches in terms of instrumentation efficiency and obturation quality can provide valuable insights for clinicians in selecting the most appropriate technique for pediatric patients [10]. Furthermore, reduced treatment time associated with rotary instrumentation can improve patient cooperation and overall treatment experience in children [11]. This is especially important in managing anxious or behaviorally challenging patients, where shorter procedures can lead to better clinical outcomes. At the same time, ensuring high-quality obturation remains paramount to prevent treatment failure and the need for retreatment or extraction [12]. Despite the growing adoption of rotary systems in pediatric dentistry, there is limited consensus regarding their superiority over traditional hand files in terms of obturation quality [13]. Variations in study design, sample size and evaluation criteria have contributed to inconsistent findings. Therefore, a systematic and comparative evaluation is necessary to establish clear evidence regarding the effectiveness of Kedo-S rotary files [14]. Therefore, it is of interest to assess the comparative efficiency and obturation quality of Kedo-S rotary files versus conventional hand files in pediatric endodontics, thereby guiding evidence-based clinical decision-making and improving treatment outcomes in primary teeth.

## Methodology:

This study was designed as a randomized controlled clinical trial conducted in the Department of Pediatric and Preventive Dentistry at a dental teaching institution. The study aimed to compare the efficiency and obturation quality of Kedo-S rotary files versus conventional hand files in primary teeth requiring pulpectomy. A total of 100 primary teeth indicated for pulpectomy in children aged 4-9 years were included in the study. The sample size was determined based on previous similar studies and statistical power analysis to ensure adequate representation and reliability of results. The selected teeth were randomly divided into two groups, with 50 teeth in each group. Children aged between 4 and 9 years with primary molars exhibiting necrotic pulp or irreversible pulpitis, having at least two-thirds of root length remaining and no excessive pathological root resorption, were included in the study, provided they were cooperative or manageable (Frankl behavior rating 3 or 4). Teeth with internal or external root resorption involving more than one-third of the root, non-restorable teeth, cases with abscess or cellulitis requiring antibiotic therapy, children with systemic diseases or special healthcare needs and previously treated primary teeth were excluded. A total of 100 teeth were randomly allocated into two groups using a computer-generated randomization method: Group I (n = 50) underwent instrumentation using Kedo-S rotary files, while Group II (n = 50) was treated with conventional stainless steel hand K-files. All clinical procedures were performed by a single trained operator to minimize variability. Pre-operative clinical and radiographic assessments were conducted to confirm diagnosis and suitability for lumpectomy. Local anesthesia was administered, followed by rubber dam isolation to maintain aseptic conditions. Standard access cavity preparation was performed using a high-speed handpiece with a sterile bur and working length was determined using an intraoral periapical radiograph, maintained 1 mm short of the radiographic apex. Canal preparation in Group I was carried out using Kedo-S rotary files according to the manufacturer's instructions with a low-speed endodontic motor, whereas Group II canals were prepared using stainless steel hand K-files with a step-back technique. During instrumentation, canals were irrigated with 1% sodium hypochlorite and saline. The canals were then dried using sterile paper points and obturated with a resorbable material such as zinc oxide eugenol or calcium hydroxide-iodoform paste using a lentulo spiral. The access cavity was restored with appropriate restorative material and stainless steel crowns were placed where indicated. The primary outcome measure was instrumentation time, recorded using a stopwatch from initial file insertion to completion of biomechanical preparation. Obturation quality was evaluated radiographically and categorized as underfilled (more than 2 mm short of apex), optimally filled (at apex or within 0-2 mm short), or overfilled (beyond apex), along with assessment of the presence or absence of voids. Radiographic evaluation was performed by two independent blinded examiners to minimize observer bias, with a third evaluator consulted in case of disagreement. Data were tabulated and analyzed using statistical software such as SPSS, with descriptive statistics used for data summarization, independent t-test applied for comparison of instrumentation time and Chi-square test used for evaluating obturation quality and voids; a p-value of <0.05 was considered statistically significant. Ethical approval was obtained from the Institutional Ethical Committee and informed consent was secured from parents or guardians of all participating children, with all procedures conducted in accordance with standard ethical guidelines for human research.

## Results:

A total of 100 primary teeth were included in the study and equally divided into two groups: Group I (Kedo-S rotary files) and Group II (hand K-files), with 50 samples in each group. All cases were completed successfully and included in the final analysis. The mean instrumentation time in Group I (Kedo-S rotary) was significantly lower compared to Group II (hand files) as shown in Table 1. This indicates greater efficiency of rotary instrumentation in pediatric endodontics. Table 2 shows that a higher percentage of optimally filled canals were observed in Group I (72%) compared to Group II (52%). The presence of voids within the obturated canals was also evaluated. As seen in Table 3, fewer voids were observed in the Kedo-S group compared to the hand file group. Chi-square test was applied to evaluate the association between instrumentation technique and obturation quality. Table 4 shows a statistically significant association between the type of instrumentation and both obturation quality and presence of voids. Statistical analysis was also performed using STATA software to validate the findings. The STATA analysis (Table 5) confirms that the use of Kedo-S rotary files significantly reduced instrumentation time and improved obturation quality, with fewer voids compared to hand files. The results of the present study indicate that Kedo-S rotary files are significantly more efficient in terms of instrumentation time and produce better obturation quality with fewer voids compared to conventional hand files. These findings were consistent across descriptive statistics, inferential tests and STATA analysis.

## Discussion:

The present study was conducted to evaluate and compare the efficiency and obturation quality of Kedo-S rotary files with conventional hand files in primary teeth. The results demonstrated that Kedo-S rotary instrumentation significantly reduced instrumentation time and yielded superior obturation quality with fewer voids compared to hand files. These findings highlight the clinical advantages of pediatric rotary systems in endodontic treatment of primary teeth. One of the most significant outcomes of this study was the marked reduction in instrumentation time in the Kedo-S group. This finding is in agreement with Jeevanandan and Govindaraju (2018), [14] who reported that Kedo-S rotary files significantly decrease chairside time compared to manual instrumentation. This reduction in time is particularly beneficial in pediatric patients, where shorter treatment duration improves cooperation and overall clinical management. Similarly, Kalita *et al.* (2021) [15] also observed that rotary instrumentation systems provide faster canal preparation due to their continuous cutting action and superior design, which aligns with the findings of the present study. In terms of obturation quality, the present study showed a higher percentage of optimally filled canals in the Kedo-S group compared to the hand file group. This result is consistent with the observations of Tofangchiha *et al.* (2022), [16] who concluded that the improved shaping ability of Kedo-S rotary files facilitates better adaptation of obturating material, resulting in more homogeneous and optimal fillings. The uniform taper created by rotary files enhances the flow of obturating material within the canal, thereby reducing the chances of underfilling or overfilling. Another important finding of the present study was the significantly lower presence of voids in the Kedo-S group. This observation is supported by Swaminathan *et al.* (2025), [17] who in their review highlighted that pediatric rotary systems ensure more predictable canal shaping and better debridement, which directly contributes to improved obturation quality with minimal voids. The enhanced cleaning efficiency of rotary instruments allows for better removal of debris and necrotic tissue, thereby improving the sealing ability of obturating materials. Furthermore, the safety and effectiveness of Kedo-S rotary files observed in the present study are in accordance with the findings of Sulaiman *et al.* (2025), [18] who reported that Kedo-S files are associated with fewer procedural errors and reduced dentinal damage compared to other instrumentation techniques. This is particularly important in primary teeth, where preservation of remaining tooth structure is crucial due to ongoing physiological root resorption and proximity to the developing permanent tooth germ. Overall, the results of the present study are in strong agreement with previous literature, reinforcing that Kedo-S rotary files provide superior efficiency and improved obturation quality compared to conventional hand files. The consistency of these findings across multiple studies supports the growing preference for pediatric rotary systems in modern endodontic practice and emphasizes their role in enhancing the quality and predictability of pulpectomy procedures in children.

## Conclusion:

Kedo-S rotary files showed significantly high efficiency by reducing instrumentation time and showed superior obturation quality with a higher percentage of optimally filled canals compared to hand files. The incidence of voids was lower in the rotary group, indicating better canal preparation and filling adaptation. Thus, data shows that Kedo-S rotary files are a reliable and effective alternative to conventional hand instrumentation, enhancing both clinical performance and treatment outcomes in primary teeth.

## Figures and Tables

**Table 1:** Comparison of mean instrumentation time (in minutes)

**Group**	**N**	**Mean ± SD**	**t-value**	**p-value**
**Group**	**N**	**Mean ± SD**	**t-value**	**p-value**
Group I (Kedo-S)	50	6.42 ± 1.15	9.87	<0.001*
Group II (Hand files)	50	9.85 ± 1.72		

**Table 2:** Comparison of obturation quality between groups

**Obturation Quality**	**Group I (Kedo-S)**	**Group II (Hand files)**	**Total**
Underfilled	6 (12%)	14 (28%)	20
Optimally filled	36 (72%)	26 (52%)	62
Overfilled	8 (16%)	10 (20%)	18
Total	50	50	100

**Table 3:** Comparison of voids in obturation

**Voids Presence**	**Group I (Kedo-S)**	**Group II (Hand files)**	**Total**
Present	9 (18%)	20 (40%)	29
Absent	41 (82%)	30 (60%)	71
Total	50	50	100

**Table 4:** Chi-square test for obturation quality

**Parameter**	**Chi-square value**	**p-value**
Obturation Quality	6.78	0.034*
Voids in Obturation	5.56	0.018*

**Table 5:** STATA output summary

**Variable**	**Coefficient**	**Std. Error**	**z-value**	**p-value**	**95% Confidence Interval**
Instrumentation Time	-3.43	0.35	-9.8	<0.001*	-4.12 to -2.74
Optimal Obturation	1.12	0.48	2.33	0.020*	0.18 to 2.06
Presence of Voids	-0.98	0.41	-2.39	0.017*	-1.78 to -0.18
